# Supplementary material for: Preparation and Application of Fe-N Co-Doped GNR@CNT Cathode Oxygen Reduction Reaction Catalyst in Microbial Fuel Cells
Source: Nanomaterials (Basel). 2021 Feb 2;11(2):377. doi: 10.3390/nano11020377 (PMC7912981; doi:10.3390/nano11020377)
Supplement: Supplementary file 1 [file nanomaterials-11-00377-s001.pdf]

nanomaterials

MDPI

Supplementray Material

Preparation and application of Fe-N co-doped GNR@CNT cathode oxygen reduction reaction catalyst in microbial fuel cells

Man Zhang, Zhaokun Ma* and **Huaihe Song***

Beijing Key Laboratory of Electrochemical Process and Technology for Materials, Beijing University of Chemical Technology, Beijing 100029, China; 2018400229@mail.buct.edu.cn

*Correspondence: mazk@mail.buct.edu.cn (Z.M.); songhh@mail.buct.edu.cn (H.S.); Tel.: +86-10-64434916 (Z.M.); +86-10-64434916 (H.S.)

Table S1. The element contents of CNT and GNR@CNT tested by Element analysis.

	C/%	O/%	H/%	N/%	S/%
**CNT**	96.88	2.54	0.40	0.07	0.11
**GNR@CNT**	59.93	38.28	1.71	0.14	0.94

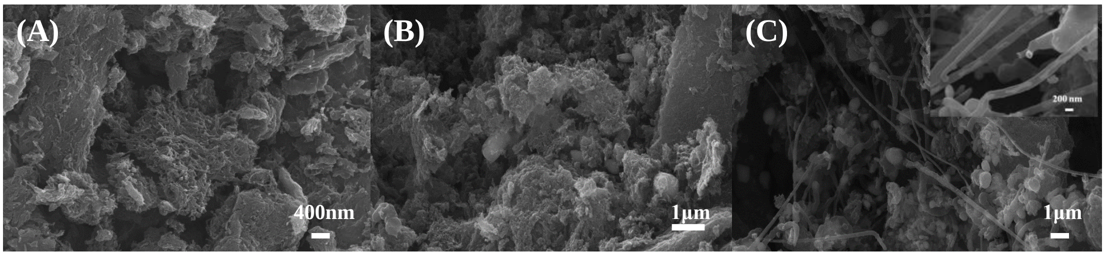

Figure S1. SEM images of (**A**) Fe-N/C-1:4:4, (**B**) Fe-N/C-1:2:2, and (**C**) Fe-N/C-1:1:1, respectively.

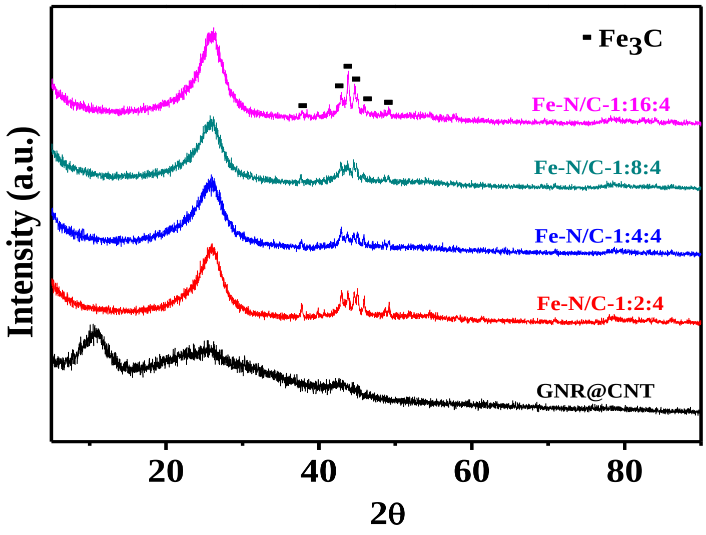

Figure S2. XRD patterns of GNR@CNT-based samples changing with the melamine-doping contents.

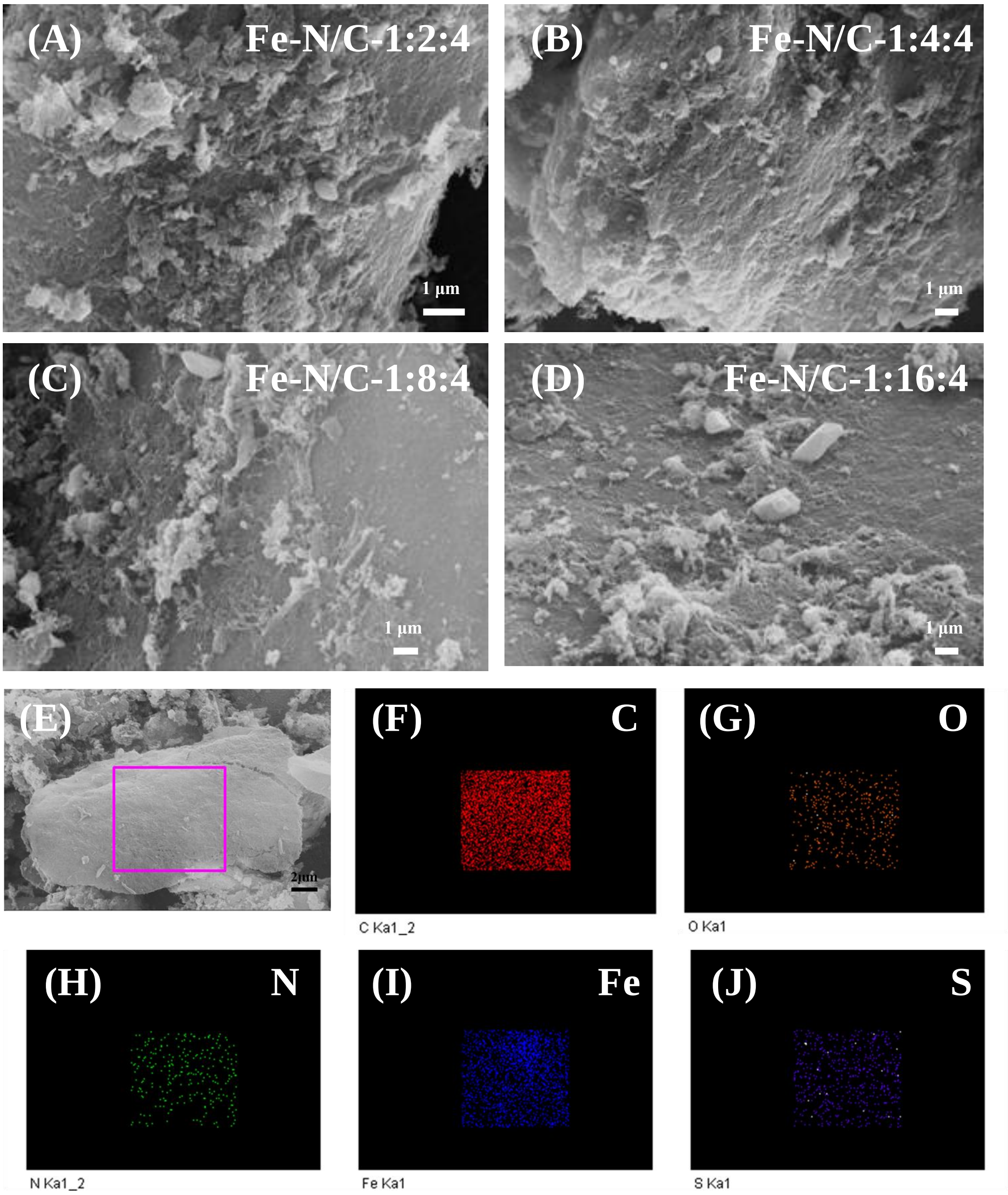

Figure S3. SEM images of (**A**) Fe-N/C-1:2:4, (**B**) Fe-N/C-1:4:4, (**C**) Fe-N/C-1:8:4, and (**D**) Fe-N/C-1:16:4, respectively. (**E**) the raw image and the EDS mapping images of (**F**) C, (**G**) O, (**H**) N, (**I**)Fe, and (**J**) S for Fe-N/C-1:4:4, respectively.

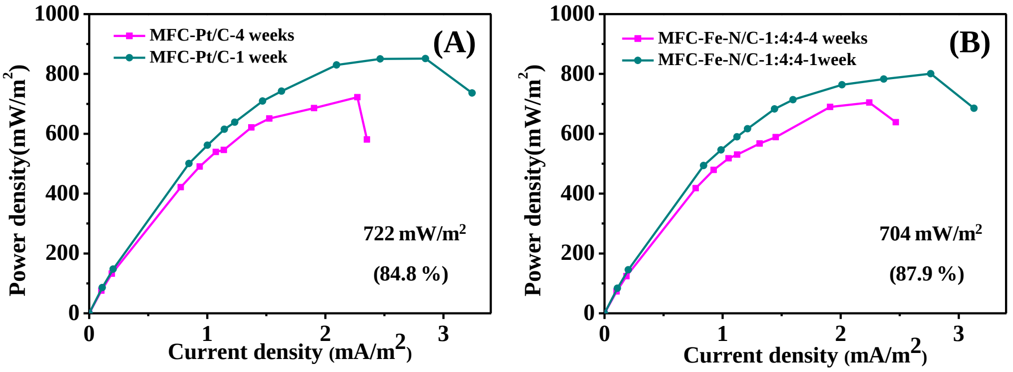

Figure S4. The power density curves of MFC-Pt/C (**A**) and MFC-Fe-N/C-1:4:4 (**B**) after cycling for 1 week and 4 weeks, respectively.

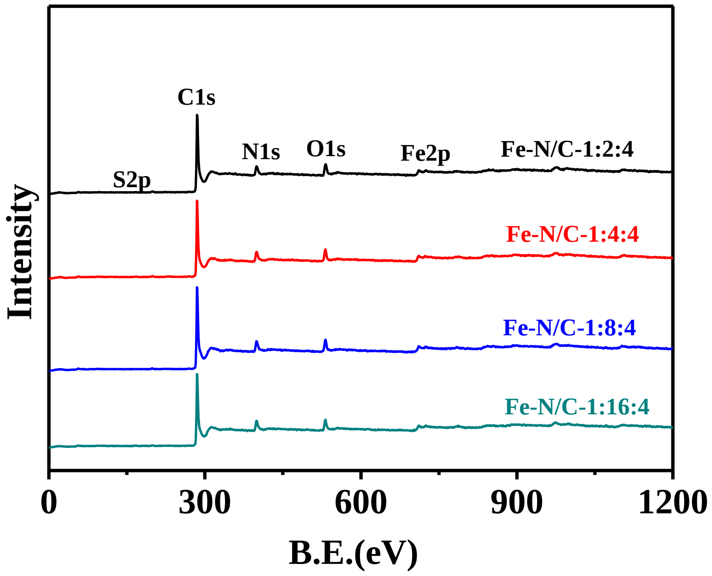

Figure S5. XPS survey spectra of Fe-N/C-1:2:4, Fe-N/C-1:4:4, Fe-N/C-1:8:4, and Fe-N/C-1:16:4 electrocatalysts, respectively.

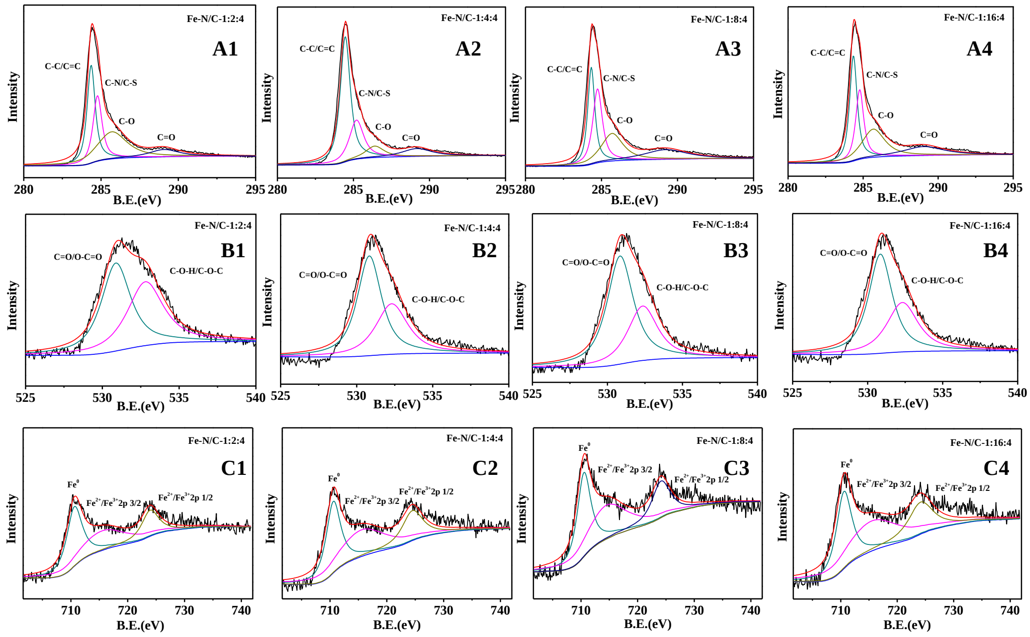

Figure S6. Deconvolution of C1s, O1s, and Fe2p XPS spectra of Fe-N/C-1:2:4, Fe-N/C-1:4:4, Fe-N/C-1:8:4, and Fe-N/C-1:16:4, respectively.

Table S2. Summary of the application of Fe-N/C as cathode catalysts in MFCs.

Carbon Source	N Source	Fe Source	Pyrolysis T(℃)	Mass(mg)	MPD	Ref
Graphene nanoribbon@ carbon nanotube	Melamine	$FeCl_3$	800	14	801 mW/m^2	This work
Active carbon	FePc	FePc	900	105	1092 mW/m^2	[1]
Polyaniline	Polyaniline	$Fe(NO_3)_3$	900	-	900 mW/m^2	[2]
Poly(vinyl alcohol)	Polypyrrole	$FeCl_3$	900	12	1400 mW/m^2	[3]
Sing-layer graphene	Melamine	$FeCl_3$	800	40	1210 mW/m^2	[4]
High conductivity graphene	Melamine	$FeCl_3$	800	40	890 mW/m^2	[4]
High activity graphene	Melamine	$FeCl_3$	800	40	1131 mW/m^2	[4]
2-methyl-imidazole	2-methyl-imidazole	$Fe(acac)_3$	1000	35	1233 mW/m^2	[5]
Polyaniline, graphene	Polyaniline	$FeCl_3$	900	40	1601 mW/m^2	[6]
Active carbon	Pyrrole	Fe_2O_3	-	20	2387 mW/m^2	[7]
Polyamide acid	Polyamide acid	$FeCl_3$	800	23	984	[8]
Bacteria	Hematite	Hematite	800	12	1928	[9]
Carbon nanotubes, polyindole	FePc	FePc	-	-	799	[10]

Reference

[1] Liu Y, Fan Y-S, Liu Z-M. Pyrolysis of iron phthalocyanine on activated carbon as highly efficient non-noble metal oxygen reduction catalyst in microbial fuel cells. Chemical Engineering Journal 2019;361:416-27.

[2] Ren P, Ci S, Ding Y, Wen Z. Molten-salt-mediated synthesis of porous Fe-containing N-doped carbon as efficient cathode catalysts for microbial fuel cells. Applied Surface Science 2019;481:1206-12.

[3] Su Y, Jiang H, Zhu Y, Zou W, Yang X, Chen J, et al. Hierarchical porous iron and nitrogen co-doped carbons as efficient oxygen reduction electrocatalysts in neutral media. Journal of Power Sources 2014;265:246-53.

[4] Wang D, Ma Z, Xie Ye, Song H. Characterization of Fe/N-doped graphene as air-cathode catalyst in microbial fuel cells. Journal of Energy Chemistry 2017;26:1187-95.

[5] Luo X, Han W, Du W, Huang Z, Jiang Y, Zhang Y. Ordered mesoporous carbon with atomically dispersed Fe-Nx as oxygen reduction reaction electrocatalyst in air-cathode microbial fuel cells. Journal of Power Sources 2020;469:228184.

[6] Cao C, Wei L, Wang G, Liu J, Zhai Q, Shen J. A polyaniline-derived iron–nitrogen–carbon nanorod network anchored on graphene as a cost-effective air-cathode electrocatalyst for microbial fuel cells. Inorganic Chemistry Frontiers 2017;4:1930-8.

[7] Zhang X, Guo X, Wang Q, Zhang R, Xu T, Liang P, et al. Iron-based clusters embedded in nitrogen doped activated carbon catalysts with superior cathodic activity in microbial fuel cells. Journal of Materials Chemistry A 2020;8:10772-8.

[8] Wang D, Ma Z, Meng X, Song H. Preparation and characterization of Fe-doped PAA as air-cathode electrocatalyst in microbial fuel cells. Catalysis Communications 2018;105:56-8.

[9] Ma X, Lei Z, Feng W, Ye Y, Feng C. Living Fe mineral@bacteria encrustation-derived and self-templated preparation of a mesoporous Fe-N-C electrocatalyst with high activity for oxygen reduction. Carbon 2017;123:481-91.

[10] Nguyen M-T, Mecheri B, Iannaci A, D'Epifanio A, Licoccia S. Iron/Polyindole-based Electrocatalysts to Enhance Oxygen Reduction in Microbial Fuel Cells. Electrochimica Acta 2016;190:388-95.
